# Epidemiological and Genetic Diversity of *Staphylococcus aureus* Causing Bloodstream Infection in Shanghai, 2009-2011

**DOI:** 10.1371/journal.pone.0072811

**Published:** 2013-09-09

**Authors:** Xu Chen, Wen-Kui Wang, Li-Zhong Han, Ying Liu, Hong Zhang, Jin Tang, Qing-Zhong Liu, Yu-Chan Huangfu, Yu-Xing Ni

**Affiliations:** 1 Department of Clinical Microbiology, Ruijin Hospital, Shanghai Jiao Tong University School of Medicine, Shanghai, China; 2 Department of Burns, Ruijin Hospital, Shanghai Jiao Tong University School of Medicine, Shanghai, China; 3 Department of Clinical Laboratory, Xinhua Hospital, Shanghai Jiao Tong University School of Medicine, Shanghai, China; 4 Department of Clinical Laboratory, Children's Hospital of Shanghai, Shanghai Jiao Tong University, Shanghai, China; 5 Department of Clinical Laboratory, Shanghai Sixth People's Hospital, Shanghai Jiao Tong University, Shanghai, China; 6 Department of Clinical Laboratory, Shanghai First People's Hospital, Shanghai Jiao Tong University, Shanghai, China; Rockefeller University, United States of America

## Abstract

**Objectives:**

*Staphylococcus aureus* or methicillin-resistant *Staphylococcus aureus* (MRSA) has been an important pathogen causing bloodstream infections. Our study aimed to investigate the epidemiological and genetic diversity of clinical *S. aureus* isolates from patients with bloodstream infection in four hospitals of Shanghai from 2009 to 2011.

**Methods:**

A collection of *S. aureus* isolates causing bloodstream infection from four hospitals in the central part of Shanghai was carried out. Antimicrobial susceptibility testings of collected isolates were performed according to the Clinical and Laboratory Standards Institute (CLSI) guidelines, and *spa*-type, multi-locus sequence typing, *agr* type and toxin gene profiling were performed to explore the molecular diversity. Moreover, MRSA strains were also characterized by Staphylococcal cassette chromosome *mec* (SCC*mec*) typing.

**Results:**

The drugs such as linezolid, teicoplanin and vancomycin were efficacious for treating *S. aureus* including MRSA bloodstream infection. Methicillin-sensitive *Staphylococcus aureus* (MSSA) strains displayed distinct diversity in molecular characterization and toxin genes, and three virulent MSSA strains encoding at least five toxins were detected. Five community-associated MRSA (CA-MRSA) strains were found, but the majority (88.7%) of MRSA strains belonged to two epidemic clones (ST239-MRSA- III and ST5-MRSA- II) with different toxin gene profiles among patients with bloodstream infection.

**Conclusions:**

Healthcare-associated MRSA (HA-MRSA) strains were still the main pathogen causing bloodstream infections in spite of the emergence of CA-MRSA strains in hospital setting.

## Introduction

*Staphylococcus aureus* or methicillin-resistant *Staphylococcus aureus* (MRSA) has been a major cause of nosocomial infections [1], and during the last two decades it increasingly causes infections in the community [2]. In the healthcare setting, *S. aureus* may cause wound infections, catheter-related infections, pneumonia, urinary tract infections and bacteremia [1], [3], while in the community it usually results in skin and soft-tissue infections (SSTIs), and occasionally necrotizing pneumonia, necrotizing fasciitis and sepsis [2]–[4]. The first case of MRSA was reported in the 1960 s, and now MRSA has reached a high prevalence of invasive infections globally. To make matter worse, patients with invasive MRSA infections such as bloodstream infection (BSI) show a high mortality [5]–[6]. Therefore, molecular epidemiology, antibiotic resistance pattern, virulence factors and clinical information of *S*. *aureus* bloodstream infection should be provided to clinicians or healthcare workers to improve prevention, control and treatment.

It is known that various MRSA clones circulate in different countries or regions, and that they differ in antimicrobial resistance pattern, molecular characterization and virulence factors [7]. A variety of genotyping methods have been developed to discriminate strains and understand the epidemiology of MRSA strains [8]. The common methods include multi-locus sequence typing (MLST), Staphylococcal cassette chromosome *mec* (SCC*mec*) typing, Staphylococcus protein A gene (*spa*) typing, accessory gene regulator (*agr*) typing and toxin gene profiling [9]–[12]. Above all, a thorough knowledge of *S*. *aureus* bloodstream infection will contribute to the clinical practice and outcome of patients.

As reported, the Brazilian or Hungarian clone (ST239) and the New York/Japan clone (ST5) were prevalent in most Asian countries, such as China, Korea and Japan [6]. Also, ST239-MRSA-III and ST5-MRSA-II clones usually cause healthcare-associated infections in China [9]–[11]. Recently, several studies have focused on the invasive MRSA infections, especially bloodstream infection [5], [6], [10]–[12]. However, in Shanghai, one of the major metropolises with a large population of residents and visitors in China, only Song et al analyzed the molecular epidemiology of 103 *S*. *aureus* isolated from blood in a hospital during six years [11]. There is a need for more data about the bloodstream infections. In the present study, *S. aureus* isolates causing bloodstream infections from four hospitals in Shanghai were collected. Antimicrobial susceptibility testing, molecular characterization and toxin gene profiling of these isolates were performed, and the relationship between epidemiological classification of bloodstream infection episodes and corresponding strain type were investigated.

## Methods

### Study design

From 2009 to 2011, clinical *S. aureus* isolates which caused a bloodstream infection were collected in four hospitals. The four hospitals (Hospital A to D) located in four different administrative districts in the central part of Shanghai, serving for a total population of around 3.3 million, approximately equivalent to fifteen percent of the whole population in Shanghai. Hospital A, B and C were comprehensive tertiary teaching hospitals, and Hospital D was a tertiary children's hospital. Totally, in these four hospitals from 2009 to 2011 *S. aureus* bloodstream infection represented a proportion of 5.7% (327 out of 5712 cases from medical records), among which 108 cases (all available cases during this period) were enrolled in this study. All these isolates were recovered to determine the antimicrobial resistance pattern, *spa*-type, sequence type (ST), *agr* type and toxin gene profiling, and the SCC*mec* type of MRSA strains were also determined to know the circulating clone. This study was approved by Ruijin Hospital Ethics Committee (Shanghai Jiao Tong University School of Medicine), and the Review Board exempted the need for informed consent because this retrospective study mainly focused on bacteria and did no interventions to patients.

### Definitions

The *S. aureus* bloodstream infection was defined by the isolation of *S. aureus* from blood cultures of patients with/without clinical signs or symptoms, such as fever, chills and sweats. The bloodstream infections were epidemiologically classified as i) healthcare-associated community-onset infection (HACOI), ii) healthcare-associated hospital-onset infection (HAHOI) and iii) community-associated infection (CAI) according the standards of the U.S. Centers for Disease Control and Prevention [5] by checking clinical data. MRSA strains were those expressing *mec*A or another mechanism of methicillin resistance such as changes in affinity of penicillin-binding proteins for oxacillin, and were classified as healthcare-associated MRSA (HA-MRSA) strain type or community-associated MRSA (CA-MRSA) strain type according to their genetic features [13]; According to the situation of Shanghai, HA-MRSA was defined as strains possessing SCC*mec* I, II or III, and CA-MRSA was defined as strains possessing SCC*mec* IV or V in this study.

### Pathogen, identification and antimicrobial susceptibility testing

Collected isolates were checked by Vitek-2 system and/or phenotypic tests as previously described [4], [9], [14], and the isolates were recovered to conduct antimicrobial susceptibility testing with the disk diffusion method according to the guidelines of Clinical and Laboratory Standards Institute (CLSI) [15]. The drugs tested included penicillin (10 units), oxacillin (1 μg), cefoxitin (30 μg), gentamicin (10 μg), tobramycin (10 μg), erythromycin (15 μg), clindamycin (2 μg), sulfamethoxazole/trimethoprim (25 μg), rifampicin (5 μg), linezolid (30 μg), mupirocin (5 μg), fusidic acid (10 μg) and teicoplanin (30 μg). Minimum inhibitory concentration (MIC) of vancomycin was determined by the agar dilution method [15]. Screening tests for β-lactamase production were performed by the penicillin zone-edge test and nitrocefin-based test if the zone diameter of penicillin indicated sensitivity, and inducible resistance to clindamycin was tested by the D-test. MRSA strains were screened by cefoxitin disk, and high-level mupirocin resistance was screened by the 200 μg mupirocin disk. *S. aureus* ATCC25923 and ATCC29213 were included for the quality control of the disk diffusion test and MIC detection respectively.

### Detecting molecular epidemiologic characters

Bacterial DNA was extracted with the simplified alkaline-lysis method [14]. MRSA strains were verified by the detection of the *mec*A gene [16], [17]. The *spa* repeat region of all isolates was amplified and sequenced [18], and the *spa*-type was gained via the online database (http://www.spaserver.ridom.de/). The sequence type (ST) was characterized by multi-locus sequence typing (MLST), and the products of seven house-keeping gene fragments were sequenced (Sangon Biotech, Shanghai) and compared with allele profiles from database of *S. aureus* (http://saureus.mlst.net/). SCC*mec* typing of MRSA strains was conducted as previously described [14].

### Grouping of *agr* allele and toxin gene profiling by polymerase chain reaction

The accessory gene regulator alleles (*agr* I-IV) were determined by a reported method [19]. Toxin gene profiles of all isolates were elucidated by detecting a variety of clinically significant toxin genes encoding staphylococcal enterotoxins (*sea-see* and *seg-sej*), exfoliative toxin (*eta* and *etb*), toxic shock syndrome toxin 1 (*tst*) and Panton-Valentine leukocidin (*pvl*) [19].

### Statistical analysis

Statistical data were processed in Excel format, and the univariate comparison was performed using the chi-square or Fisher's exact test as appropriate. All statistical analysis was conducted by SAS 8.2 (SAS Institute Inc., Cary, NC, USA). It was considered statistically significant if the two-sided P-value <0.05.

## Results

### Collected isolates and classification of bloodstream infection

A total of 108 non-duplicated *S. aureus* isolates from 108 patients with bloodstream infection (one isolate from one patient) were enrolled in this study, including 54 from Hospital A, 12 from Hospital B, 26 from Hospital C, 16 from Hospital D. By retrospectively reviewing clinical data, of these 108 BSI episodes 100 episodes were defined as healthcare-associated infections including 77 episodes as healthcare-associated hospital-onset infection (HAHOI) and 23 episodes as healthcare-associated community-onset infection (HACOI), and 8 episodes were defined as community-associated infection (CAI) according the standards of the U.S. Centers for Disease Control and Prevention as previously described [5].

### Antimicrobial susceptibility testing

Sixty-two (57.4%) out of 108 *S. aureus* isolates were identified as MRSA by cefoxitin disk screening and *mec*A gene confirming. As shown in Table 1, all isolates were sensitive to linezolid, teicoplanin and vancomycin. Among MSSA and MRSA the minimum inhibitory concentration (MIC) of vancomycin both ranged from 0.5–2 μg/ml, meanwhile MIC_50_ and MIC_90_ values of MSSA were both 1 μg/ml, which were the same as those of MRSA (Table 1). Only 3 isolates were sensitive to penicillin with β-lactamase negative. The rates of resistance to other drugs varied from 11.1% to 62.0%. Moreover, inducible resistance to clindamycin was found among 6 isolates (5.6%) by the D-test, and also 7 isolates were showing high-level mupirocin resistance (Details in Table 1).

**Table 1:** The antibiotic resistance rate of 108 isolates in this study.

Drug	Resistance rate (%)			P-value
	Overall	MSSA	MRSA	
Penicillin	97.2	93.5	100	0.1478
Oxacillin	57.4	0	100	–
Cefoxitin	57.4	0	100	–
Gentamicin	51.9	6.5	85.5	<0.0001
Tobramycin	62	28.3	87.1	<0.0001
Erythromycin	62	32.6	82.3	<0.0001
Clindamycin^a^	52.8	17.4	77.4	<0.0001
Sulfamethoxazole/ trimethoprim	16.7	8.7	22.6	0.0555
Rifampicin	37	19.6	50	0.0012
Linezolid	0	0	0	–
Mupirocin^b^	19.4	19.6	19.4	0.9782
Fusidic acid	11.1	15.2	8.1	0.2422
Teicoplanin	0	0	0	–
Vancomycin^c^	0	0	0	–

a : 6 isolates (4 MSSA and 2 MRSA) inducible resistance to clindamycin.

b : 7 isolates (1 MSSA and 6 MRSA) showing high-level mupirocin resistance.

c : MIC range, 0.5∼2 μg/ml; 10 isolates (5 MSSA and 5 MRSA) MIC = 0.5 μg/ml; 89 isolates (38 MSSA and 51 MRSA) MIC = 1 μg/ml; 9 isolates (3 MSSA and 6 MRSA) MIC = 2 μg/ml.

P-value, two-sided P-value calculated by the chi-square or Fisher's exact test as appropriate.

### Strain type and its relationship with classification of BSI

Among 62 (57.4%) *mec*A-positive MRSA, the SCC*mec* typing revealed four types of SCC*mec* including SCC*mec* II (18, 29.0%), SCC*mec* III (39, 62.9%), SCC*mec* IV (2, 3.2%) and SCC*mec* V (3, 4.8%), thus indicating that 5 CA-MRSA strains and 57 HA-MRSA strains were detected according to genetic features as described previously. Furthermore, the relationship between epidemiological classification of 108 bloodstream infection episodes and corresponding strain type was explored and displayed in Table 2. HAHOI episodes were caused by 52 HA-MRSA strains, 1 CA-MRSA strain and 24 MSSA strains, while HACOI episodes were brought by 5 HA-MRSA strains, 3 CA-MRSA strains and 15 MSSA strains, respectively. CAI was found to be caused by 1 CA-MRSA strain and 7 MSSA strains.

**Table 2:** The relationship between epidemiological classification of bloodstream infection and strain type.

Epidemiological classification	Strain type		
Healthcare-associated	HA-MRSA	CA-MRSA	MSSA
Hospital-onset infection (HAHOI)	52	1	24
Community-onset infection (HACOI)	5	3	15
Community-associated infection (CAI)	0	1	7

HA-MRSA, healthcare-associated methicillin-resistant *Staphylococcus aureus.*

CA-MRSA, community-associated methicillin-resistant *Staphylococcus aureus.*

MSSA, methicillin-sensitive *Staphylococcus aureus*.

### Molecular epidemiological characteristics and toxin genes

A total of 39 different *spa*-types and 23 sequence types (STs) were found among all isolates. Grouping of *agr* allele indicated that *agr* I to IV was detected in 68, 27, 6 and 2 isolates respectively, and 5 isolates were negative for all the four *agr* alleles. By detecting 13 kinds of clinically significant toxin genes, the prevalence of these genes among *S. aureus* isolates was obtained (Table 3). The *sei*, *seg*, *sea* genes were most prevalent (37.0%, 25.0% and 20.4%, respectively), and only one MSSA isolate was found both *eta*- and *etb*-positive. Thirteen (12.0%) isolates including 3 MSSA and 10 MRSA carried a *tst* gene, and three isolates (2 MSSA and 1 MRSA) were found to be *pvl*-positive. One toxin gene (*see*) was not detected among all collected isolates, and other four toxin genes (*sed*, *sej*, *eta* and *etb*) were not found in the MRSA strains. Statistical analysis suggested that there was no significant difference among detected toxin genes between MSSA and MRSA strains.

**Table 3:** Prevalence of toxin genes among *S. aureus* causing bloodstream infection.

Toxin gene	No. of positive isolates (% of 108)	No. distributing in		P-value
		MSSA (n = 46)	MRSA (n = 62)	
*sea*	22 (20.4)	7	15	0.2521
*seb*	7 (6.5)	2	5	0.7035
*sec*	16 (14.8)	8	8	0.5162
*sed*	2 (1.9)	2	0	0.1791
*see*	0 (0.0)	0	0	–
*seg*	27 (25.0)	11	16	0.8222
*seh*	7 (6.5)	3	4	1.0000
*sei*	40 (37.0)	15	25	0.4117
*sej*	2 (1.9)	2	0	0.1791
*eta*	1 (0.9)	1	0	0.4259
*etb*	1 (0.9)	1	0	0.4259
*tst*	13 (12.0)	3	10	0.1292
*pvl*	3 (2.8)	2	1	0.7924

*sea-see* and *seg-sej*, gene encoding staphylococcal enterotoxins SEA-SEE and SEG-SEJ; *eta* and *etb*, gene encoding exfoliative toxin A and B; *tst*, gene encoding toxic shock syndrome toxin 1; *pvl*, gene encoding Panton-Valentine leukocidin. P-value, two-sided P-value calculated by the chi-square or Fisher's exact test as appropriate.

The combinative data of phenotypic resistance pattern, *spa*-type, sequence type (ST), *agr* allele and toxin gene profile of MSSA strains indicated that MSSA exhibited great diversity in both genotypes and toxin genes (Table 4). Thirty *spa*-types were detected in 46 MSSA strains, and nearly one combination of phenotypic resistance pattern, *spa*-type, *agr* allele and toxin gene profile corresponded to one strain. Still, three MSSA strains (ST30-t318, ST5-t045 and ST5-t306) were more outstanding by encoding at least five toxins. Meanwhile, one *eta*- and *etb*-positive MSSA strain (a new *spa*-type designated t11685, ST2073 with *agr* IV) was found (Table 4).

**Table 4:** Phenotypic resistance pattern, *spa*-type, sequence type (ST), *agr* allele and toxin gene profiles among MSSA strains.

Phenotypic resistance pattern	*spa*-type	ST	*agr* allele	Toxin gene profile	No. of strains
None	t267	97	I	*none*	1
None	t11686	1956	IV	*none*	1
E,CC	t321	1	III	*sea,sec,seh*	1
P	t062	5	II	*seg,sei*	1
P	t078	25	I	*seb,sei*	1
P	t084	15	II	*none*	1
P	t164	20	I	*sei*	2
P	t189	188	I	*seb*	1
P	t195	20	I	*sei*	1
P	t304	6	I	*sea*	1
P	**t318**	**30**	III	*sec,seg,sei,tst,pvl*	1
P	t701	6	I	*sea*	1
P	t11687	573	II	*sec,sei*	1
P	NT	1667	NT	*none*	1
P,CC,FD	t5554	630	I	*none*	1
P,CN,TOB,E,CC	t571	398	I	*none*	1
P,CN,TOB,E,SXT	t037	241	I	*sea*	1
P,E	t034	2077	I	*pvl*	1
P,E	t164	20	I	*sei*	1
P,E	t491	15	II	*none*	1
P,E	t571	2077	I	*none*	1
P,E,CC	t11685	2073	IV	*seg,sei,eta,etb*	1
P,E,CC,MUP,FD	t377	630	I	*none*	1
P,E,CC,RA	t127	1	III	*sec,seh*	1
P,E,RA	t1250	398	I	*none*	1
P,E,RA,FD	**t045**	**5**	II	*sea,sec,seg,sei,tst*	1
P,E,SXT,RA,MUP,FD	t803	15	II	*none*	1
P,FD	t377	630	I	*none*	1
P,MUP	t034	398	I	*none*	1
P,RA,MUP	NT	30	III	*sec,seg,sei,tst*	1
P,RA,MUP	t062	5	II	*seg,sei*	1
P,RA,MUP,FD	t548	5	II	*sed,seg,sei,sej*	1
P,SXT,RA,MUP	t377	1821	I	*none*	1
P,SXT,RA,MUP,FD	t1346	72	I	*seg,sei*	1
P,TOB	t084	15	II	*none*	1
P,TOB	t091	7	I	*none*	2
P,TOB	t091	306	I	*sea*	1
P,TOB	t2616	7	I	*none*	1
P,TOB	t3386	630	I	*none*	1
P,TOB	t377	630	I	*none*	1
P,TOB,E	t091	7	I	*sea*	1
P,TOB,E	**t306**	**5**	II	*sec,sed,seg,sei,sej*	1
P,TOB,E,CC	t571	398	I	*none*	1
P,TOB,MUP	t127	1	III	*sec,seh*	1
**Total**					46

P, penicillin (10 units); OX, oxacillin (1 μg); CX, cefoxitin (30 μg); CN, gentamicin (10 μg); TOB, tobramycin (10 μg); E, erythromycin (15 μg); CC, clindamycin (2 μg); SXT, sulfamethoxazole/trimethoprim (25 μg); RA, rifampicin (5 μg); LZD, linezolid (30 μg); MUP, mupirocin (5 μg); FD, fusidic acid (10 μg); None, sensitive to all tested drugs.

*spa*, Staphylococcus protein A gene; *agr*, accessory gene regulator; NT, not-typeable; *sea-see* and *seg-sej*, gene encoding staphylococcal enterotoxins SEA-SEE and SEG-SEJ; *eta* and *etb*, gene encoding exfoliative toxin A and B; *tst*, gene encoding toxic shock syndrome toxin 1; *pvl*, gene encoding Panton-Valentine leukocidin; *none*, no detection of above toxin genes.

And in Table 5, the phenotypic resistance pattern indicated that 61 out of 62 (98.4%) MRSA strains were resistant to at least three classes of antibiotics, which were termed multi-drug resistance. To further explore epidemiological MRSA clones among patients with bloodstream infection, analysis of combined molecular data showed nine MRSA clones circulating among patients with bloodstream infections and that 88.7% of MRSA isolates belonged to two prevalent MRSA clones (ST239-MRSA-III and ST5-MRSA-II). Interestingly, the two major clones were prevalent among Hospital A-C except Hospital D, and diverse MRSA clones (ST59-MRSA-II, ST239-MRSA-III, ST5-MRSA-IV and ST7-MRSA-V) were observed in the paediatric Hospital D. In five CA-MRSA strains/clones detected in this study, only one was found *pvl*-positive, which was t437-ST59-MRSA-V (*agr* I). Given the toxin gene profile, MRSA strains harboring SCC*mec* II mainly secreted enterotoxin G and I, while those harboring SCC*mec* III were apt to express enterotoxin A and I. Worth to be noted, among MRSA the *tst* gene was only found in t002-ST5-MRSA-II strains, which belonged to a epidemic clone from patients with BSI.

**Table 5:** Epidemiological MRSA clones among patients with bloodstream infection.

ST1-SCC*mec*II/t127 (1)	Phenotypic resistance pattern	*agr* allele	Toxin gene profile	No. of strains
ST239-SCC*mec*III/t030 (22)	P,OX,CX	III	*sec,seh*	1
	P,OX,CX,CN,TOB,E,CC,RA	I	*none*	8
	P,OX,CX,CN,TOB,E,CC,RA	I	*sea*	1
	P,OX,CX,CN,TOB,E,CC,RA	I	*sea,sei*	1
	P,OX,CX,CN,TOB,E,CC,RA,MUP	I	*none*	1
	P,OX,CX,CN,TOB,E,RA	I	*none*	1
	P,OX,CX,CN,TOB,RA	I	*none*	1
	P,OX,CX,CN,TOB,RA	I	*sea*	1
	P,OX,CX,CN,TOB,RA	I	*sea,sei*	4
	P,OX,CX,CN,TOB,RA	I	*seg*	1
	P,OX,CX,CN,TOB,RA,FD	I	*none*	1
	P,OX,CX,CN,TOB,RA,MUP	I	*sea*	1
ST239-SCC*mec*III/t037 (13)	P,OX,CX,E,CC	I	*none*	1
	P,OX,CX,CN,TOB,E,CC	I	*sea,seh*	1
	P,OX,CX,CN,TOB,E,CC,SXT	I	*none*	3
	P,OX,CX,CN,TOB,E,CC,SXT	I	*sea,sei*	3
	P,OX,CX,CN,TOB,E,CC,SXT	NT	*none*	2
	P,OX,CX,CN,TOB,E,CC,SXT,FD	I	*none*	1
	P,OX,CX,CN,TOB,E,CC,SXT,FD	I	*sea*	1
	P,OX,CX,CN,TOB,E,CC,SXT,MUP	I	*none*	1
ST239-SCC*mec*III/t129 (1)	P,OX,CX,CN,TOB,E,CC,SXT,MUP	I	*sea,seh*	1
ST239-SCC*mec*III/t459 (3)	P,OX,CX,CN,TOB,E,CC,RA	I	*none*	1
ST5-SCC*mec*II/t002 (14)	P,OX,CX,CN,TOB,E,CC,RA	I	*none*	3
	P,OX,CX,CN,TOB,E,CC	II	*seb,seg,sei*	1
	P,OX,CX,CN,TOB,E,CC	II	*sec,seg,sei,tst*	2
	P,OX,CX,CN,TOB,E,CC	II	*seg,sei*	1
	P,OX,CX,CN,TOB,E,CC	II	*seg,sei,tst*	4
	P,OX,CX,CN,TOB,E,CC	NT	*sec*	1
	P,OX,CX,CN,TOB,E,CC,MUP	II	*sec,seg,sei,tst*	2
	P,OX,CX,CN,TOB,E,CC,SXT,RA	II	*sec,seg,sei,tst*	1
	P,OX,CX,E,CC	II	*seb,seg,sei*	1
	P,OX,CX,TOB,E,CC,SXT,RA,MUP	II	*sec,seg,sei,tst*	1
ST5-SCC*mec*II/t463 (1)	P,OX,CX,CN,TOB,E,RA,FD	II	*sei*	1
ST5-SCC*mec*II/t11684 (1)	P,OX,CX,CN,TOB,E,CC	II	*seg,sei*	1
ST59-SCC*mec*II/t163 (1)	P,OX,CX,E,CC,RA,MUP,FD	I	*seb*	1
ST59-SCC*mec*IV/t172 (1)	P,OX,CX,E,CC,MUP	I	*sea,seb,seh*	1
ST5-SCC*mec*IV/t062 (1)	P,OX,CX,E,RA,MUP	II	*sei*	1
ST5-SCC*mec*V/t062 (1)	P,OX,CX,CN,TOB,E,CC,MUP	II	*seg,sei*	1
ST7-SCC*mec*V/t091 (1)	P,OX,CX,RA,MUP	NT	*none*	1
ST59-SCC*mec*V/t437 (1)	P,OX,CX,E,CC	I	*seb,pvl*	1

Clone, ST-SCC*mec*; ST, sequence type by multi-locus sequence typing; SCC*mec*, Staphylococcal cassette chromosome *mec*; *spa*, Staphylococcus protein A gene; *agr*, accessory gene regulator; NT, not-typeable.

P, penicillin (10 units); OX, oxacillin (1 μg); CX, cefoxitin (30 μg); CN, gentamicin (10 μg); TOB, tobramycin (10 μg); E, erythromycin (15 μg); CC, clindamycin (2 μg); SXT, sulfamethoxazole/trimethoprim (25 μg); RA, rifampicin (5 μg); LZD, linezolid (30 μg); MUP, mupirocin (5 μg); FD, fusidic acid (10 μg).

*sea-see* and *seg-sej*, gene encoding staphylococcal enterotoxins SEA-SEE and SEG-SEJ; *eta* and *etb*, gene encoding exfoliative toxin A and B; *tst*, gene encoding toxic shock syndrome toxin 1; *pvl*, gene encoding Panton-Valentine leukocidin; *none*, no detection of above toxin genes.

## Discussion

*Staphylococcus aureus* or methicillin-resistant *Staphylococcus aureus* has been a leading cause of infections in both hospital and community settings, and bloodstream infection caused by MRSA was one of the most serious infections with high mortality [20]–[22]. In China, *S. aureus* was one of the leading pathogens isolated from bloodstream, and the average prevalence of MRSA among *S. aureus* BSI isolates reached up to 40% [10]–[11]. Among the four hospitals included in this study the prevalence of *S. aureus* from patients with bloodstream infection was about 6%, and the high percentage of multi-drug resistant MRSA isolated from blood warned narrow spectrum of antibiotics for empirical treatment. Antimicrobial susceptibility test of all collected isolates suggested that linezolid, teicoplanin and vancomycin were efficacious drugs for treating *S. aureus* including MRSA bloodstream infections. However, by reviewing clinical data, the drugs used as empirical treatment for bloodstream infections displayed great diversity according to different physicians from varied wards or hospitals. Glycopeptides such as vancomycin were still the most widely used antibiotic for confirmed MRSA bloodstream infection, as recommended by the IDSA guidelines [23]. Despite sequential reports on linezolid-resistant *S. aureus* [24]–[26], no isolates were found to be resistant to linezolid in our study, implying that linezolid may be still effective for treating MRSA infections in Shanghai. On other hand, linezolid resistance should be monitored in case of the emergency.

Exploration of the relationship between epidemiological classification of 108 bloodstream infection episodes and corresponding strain type revealed that HA-MRSA strains were still the main pathogen causing healthcare-associated hospital-onset infection (HAHOI), and only one episode of HAHOI was found to be caused by CA-MRSA, suggesting CA-MRSA was not prevalent in hospitals for all its emergence in hospital settings. MSSA strains could lead to both healthcare-associated and community-associated infections. Nevertheless, MSSA strains displayed great diversity in both genotypes and toxin genes. By detection of the *mec*A gene, 62 MRSA strains were found. Molecular characterization analysis was further performed to shed light on the molecular epidemiology of MRSA strains causing bloodstream infection. Five CA-MRSA strains (2 SCC*mec* IV and 3 SCC*mec* V) were found, which meant CA-MRSA strains emerged in hospital setting in Shanghai, and the five CA-MRSA strains showed genetic diversity in clones (ST59-MRSA-IV, ST5-MRSA- IV, ST5-MRSA-V, ST59-MRSA-V and ST7-MRSA-V). The main genotype among SCC*mec* II strains was ST5 (t002, *agr*- IIII), whilst ST239 (t030 or t037, *agr*-I) was the main genotype among SCC*mec* III strains. Song et al analyzed the 103 *S*. *aureus* isolated from blood in a hospital of Shanghai during six years, and concluded that the percentage of ST5 and ST239 decreased among MRSA strains and that of new MSSA clonal types increased [11]. Nevertheless, the predominance of two MRSA clones (ST239-MRSA- III and ST5-MRSA- II) was observed in this study, which was consistent with a recent study in Taiwan [27]. Li et al reported that a new mobile genetic element-encoded gene (*sasX*) played an important role in MRSA epidemic (especially ST239 clone) and *sasX* acted as a virulence determinant [28]. By checking the carriage of this new gene, we found that 15 out of 108 isolates possessed the *sasX* gene. Moreover, of the 15 *sasX*-positive isolates, 13 isolates belonged to ST239 MRSA, which confirmed the report by Li et al [28]. Virulence gene profiling of MRSA strains showed that five kinds of toxin genes (*sed*, *see*, *sej*, *eta* and *etb*) were not found. Notablely, the *tst* gene responsible for fatal toxic shock syndrome (TSS) was only found in t002-ST5-SCC*mec* II MRSA strains, suggesting that patients infected with t002-ST5-SCC*mec* II MRSA may have a greater potential for developing TSS.

In the present study about one third (108/327) of *S. aureus* from 2009 to 2011 were included, and the isolates was not proportional to the number of cases in each hospital, therefore accurate prevalence rate of MRSA bloodstream infection cannot be concluded owing to the potential bias. Our study just illustrated the overall bloodstream infection caused by *S*. *aureus* or MRSA in the central part of Shanghai; two main MRSA clones (ST239-MRSA-III and ST5-MRSA-II) were prevalent among patients with bloodstream infections, whilst five CA-MRSA clones (ST59-MRSA-IV, ST5-MRSA-IV, ST5-MRSA-V, ST59-MRSA-V and ST7-MRSA-V) were found. HA-MRSA strains were still the main pathogen causing healthcare-associated bloodstream infections, despite the emergence of CA-MRSA strains in hospital setting. The antibiotics such as linezolid, teicoplanin and vancomycin were efficacious drugs for treating *S. aureus* including MRSA bloodstream infections.
